# A historical perspective on the development of antisense oligonucleotide treatments for Duchenne muscular dystrophy and spinal muscular atrophy

**DOI:** 10.1177/22143602251317422

**Published:** 2025-03-04

**Authors:** Annemieke Aartsma-Rus, Shin'ichi Takeda

**Affiliations:** 1 Department of Human Genetics, Leiden University Medical Center, Leiden, the Netherlands; 2 National Institute of Neuroscience, National Center of Neurology and Psychiatry, Kodaira, Tokyo, Japan

**Keywords:** RNA therapy, muscle, exon skipping, splice modulation

## Abstract

Splice modulating antisense oligonucleotides (ASOs) have been approved for the treatment of spinal muscular atrophy (nusinersen) and Duchenne muscular dystrophy (eteplirsen) since 2016. Nusinersen obtained full approval based on convincing functional evidence in treated patients. The treatment is currently approved in over 40 countries. By contrast, eteplirsen received accelerated approval and functional evidence from clinical trials that treatment slows down disease progression is still lacking. Approval and access is restricted to the USA and several countries in the Middle-East. In this historical perspective we look back to the development paths of these two ASOs focusing on the differences between the approaches, the target tissues and the diseases. Based on this we propose learnings for future development of ASOs for progressive neuromuscular diseases.

## Introduction

In 2016, the first two splice modulating antisense oligonucleotides (ASOs) were approved by the Food and Drug Administration (FDA, USA): eteplirsen for the treatment of Duchenne muscular dystrophy (DMD) patients with eligible pathogenic variants, and nusinersen for all types of spinal muscular atrophy (SMA).^1,2^ In the past eight years, thousands of DMD and SMA patients have been treated and real world evidence has been collected.^3–5^ For SMA, nusinersen treatment has resulted in a notable change in the natural history of the disease that in the more severe form usually was lethal before the age of 2 years.^4–7^ For DMD, on the other hand, eteplirsen received accelerated approval, pending confirmatory data to show functional benefit of treatment in patients. Thus far, lacking the confirmatory data, there is still no full approval for eteplirsen and no approval at all in Europe.^8^

In this perspective paper we want to look back at the development paths for these treatments with today's knowledge on efficacy, also taking into account the differences between disease pathology and tissues affected, to distill learnings for future ASO developments for neuromuscular diseases. We are aware of the huge effort that went into the design, optimization, preclinical and clinical development of these treatments, as well as all the elucidating the disease background and setting up the clinical infrastructures required. For this historical perspective we have had to focus. We apologize to those authors not mentioned or cited, whose contributions were nevertheless crucial for nusinersen and eteplirsen development.

## Antisense oligonucleotides for Duchenne muscular dystrophy and spinal muscular atrophy

ASOs are short, synthetic, chemically modified pieces of RNA or DNA. They hybridize to RNA transcripts via Watson-Crick basepairing and can elicit cleavage of a target transcripts, or they can modulate splicing by interfering with splicing signals.^9^ For DMD and SMA the splice modulation ASO modality is used, which requires fully modified ASOs.

Both DMD and SMA are neuromuscular disorders that lead to progressive loss of muscle function and both have splice modulating ASOs approved. However, despite these similarities there are also many differences with regards to pathology, the main target tissues and the expected molecular and treatment effects from ASO treatment (see Table 1).

**Table 1.** Characteristics of antisense oligonucleotide treatments for Duchenne muscular dystrophy and spinal muscular atrophy.

Disease	Duchenne muscular dystrophy	Spinal muscular atrophy
Approved ASO	Eteplirsen	Nusinersen
Affected tissue (therapeutic target in bold)	**Skeletal muscle**, heart, smooth muscle, brain	**Motorneurons**
Mechanism of action	Restoration of the reading frame of dystrophin transcripts	Increase exon 7 inclusion in SMN2 transcripts
Effect ASO	Production of a partially functional dystrophin as found in Becker muscular dystrophy patients	Increased levels of fully functional SMN protein
Animal models used in preclinical studies	*Mdx* mouse, requiring exon 23 skipping to restore dystrophin; humanized *mdx* hDMD/*mdx*) mouse model to test eteplirsen-induced exon skipping after local injection into healthy muscle	Models with different levels of severity with mutations in mouse *Smn1* genes and containing copies of human *SMN2* genes
Translation preclinical to clinical study	ASO used in humans different from ASO used in PK/PD* studies in *mdx* mice, dystrophin produced different from those produced in *mdx* mouse	Same ASO used in preclinical and clinical trials
Chemistry	Phosphorodiamidate morpholino oligomer	2’*O*-methoxyethyl phosphorothioate
Type of approval	Accelerated approval (FDA)	Full approval FDA, EMA and many other jurisdictions
Applicability	Patients with a pathogenic variant that requires exon 51 skipping to restore the reading frame (∼14% of patients)	All types of SMA
Route of administration	Intravenous	Intrathecal
Dosing regimen	Weekly dosing (30 mg/kg)	4 loading doses, followed by maintenance dosing every 4 months
List price	$ 300,000 per year (based on a 25 kg patient)	$125,000 per injection

*PK/PD pharmacokinetic, pharmacodynamic.

### Spinal muscular atrophy

SMA is caused by pathogenic variants involving the *SMN1* gene that encodes the SMN protein, which is crucial for motoneuron survival. SMN protein is normally produced from both *SMN1* and *SMN2* genes, where the *SMN1* gene copies contribute to ∼90% of the protein amount, as in SMN2 transcripts, exon 7 is often not included due to a few sequence differences between the *SMN1* and *SMN2* genes.^10^ This is not problematic, unless the *SMN1* genes are unable to produce SMN proteins, as is the case in SMA patients, where motoneurons rely only on the amount of SMN protein produced by the *SMN2* gene copies for survival. With insufficient amount of SMN protein, motoneurons progressively die, leading to loss of muscle function and premature death for the more severe types. Interestingly, there is copy number variation for the *SMN2* gene and there is an inverse correlation between the number of *SMN2* copies and the severity of SMA.^11^ The most severe patients (type 0) die in utero, while SMA type 1 patients have symptom onset before the age of 6 months and generally die or require terminal ventilation before the age of 2. Without treatment, these patients never reach milestones such as independent sitting, standing and walking. SMA type 2 and 3 and 4 are later onset diseases, where especially type 2 and type 3 are progressive, debilitating diseases, and only milder compared to the severe types 0 and type 1 disease,.^12^

**Figure 1.**
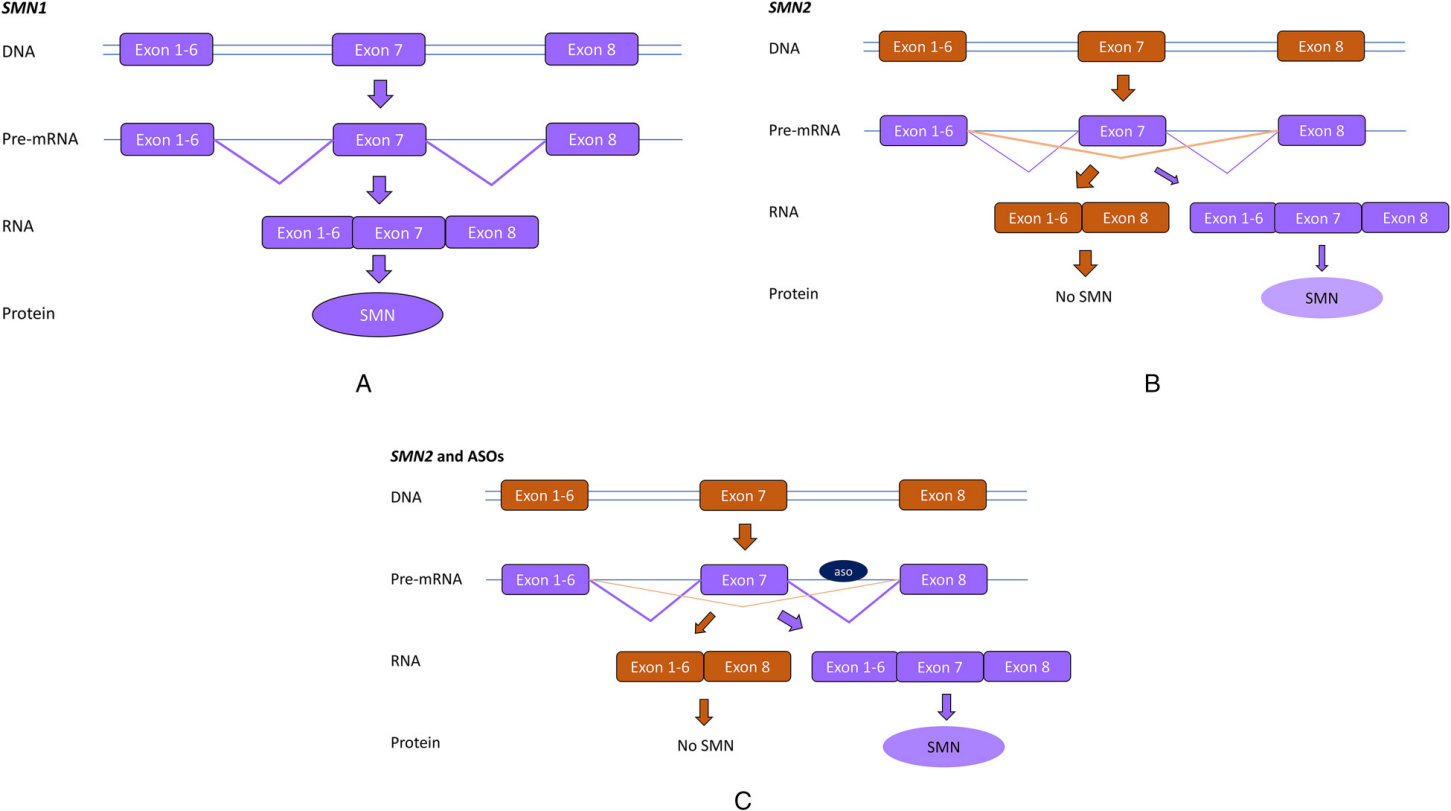
Antisense oligonucleotide treatment for spinal muscular atrophy. A. The *SMN1* gene contains 8 exons, which are spliced into an mRNA and translated into the SMN protein. B. The *SMN2* gene contains 8 exons, but exon 7 is poorly recognized by the splicing machinery. The majority of SMN2 transcripts will lack exon 7 and will be translated into an instable, non functional SMN protein. In a few transcripts, exon 7 will be included and these transcripts will be translated into SMN protein. C. Antisense oligonucleotides (ASO) targeting intron 7 of the SMN2 pre-mRNA transcripts, improve exon 7 inclusion into SMN2 mRNAs, thus increasing levels of SMN protein produced from *SMN2* genes.

As the *SMN2* gene can make an SMN protein that is identical to the one produced by *SMN1* genes, and as all SMA patients carry *SMN2* copies, the ASO splice modulating approach aims to increase the production of SMN protein from the *SMN2* gene by increasing the amount of exon 7 into SMN2 transcripts (Figure 1). This can be achieved by blocking an intronic region in intron 7 that is involved in hiding exon 7 from the splicing machinery.^10,13^

### Duchenne muscular dystrophy

DMD is caused by pathogenic variants in the *DMD* gene that disrupt the open reading frame of dystrophin transcripts. This results in a premature stop of protein translation and a non functional dystrophin protein. Normally, dystrophin prevents contraction induced damage of muscle fibers by connecting the contractile F-actin cytoskeleton to the extracellular matrix surrounding each muscle fiber. Lacking dystrophins, muscle fibers will accumulate damage, which eventually leads to patients losing skeletal muscle tissue and function.^14^ With good multidisciplinary management, most patients are wheelchair-dependent by the age of 12, need assisted ventilation by the age of 20 and die in the 2^nd^ to 4^th^ decade of life. Dystrophin also fulfills functions in the heart, smooth muscle and the brain, but the focus of dystrophin restoring approaches has been on skeletal muscle.^14^

**Figure 2.**
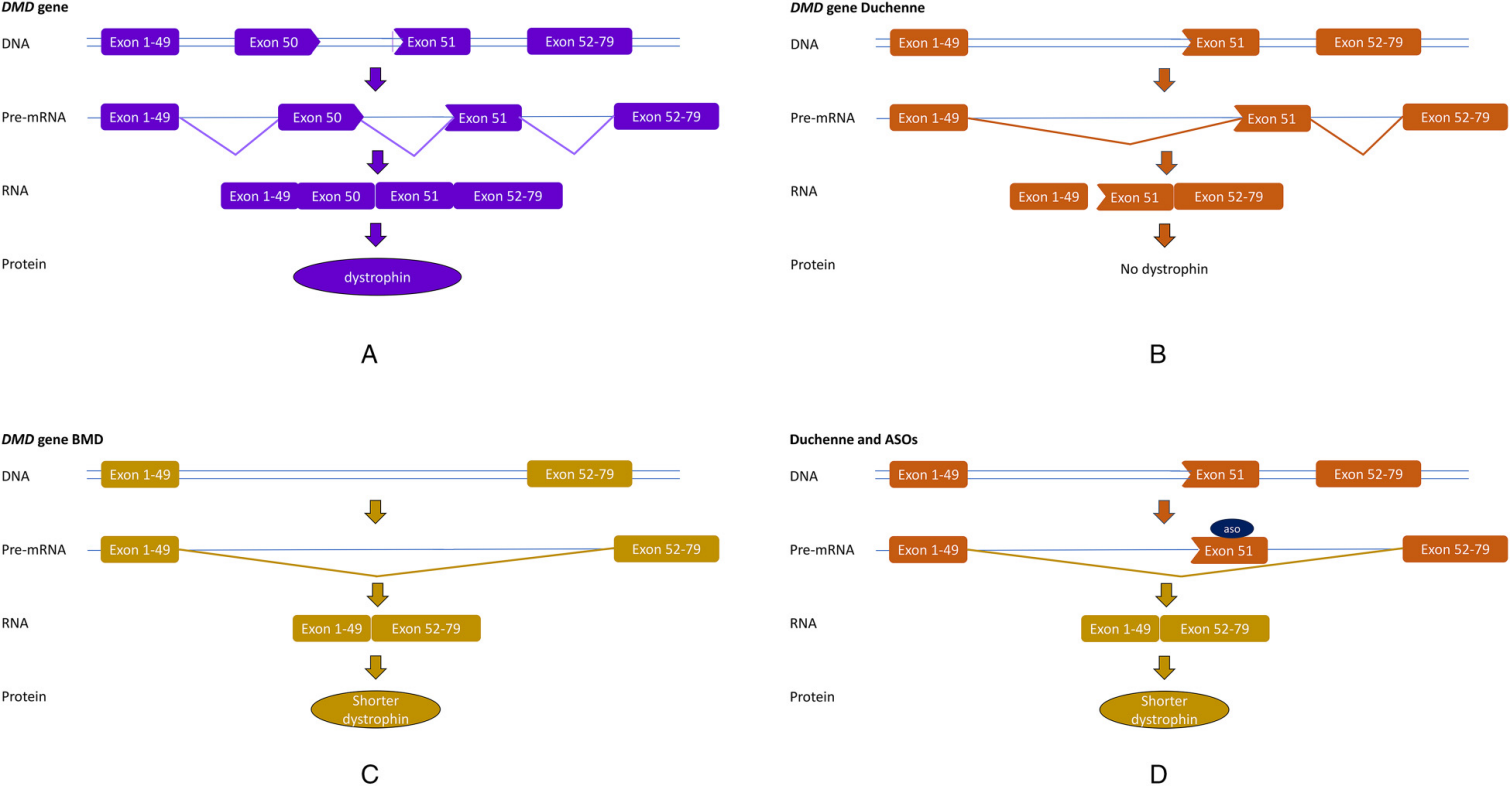
Antisense oligonucleotide treatment for Duchenne muscular dystrophy. A. The DMD gene contains 79 exons, which are spliced into an mRNA that is translated into the dystrophin protein. B. In Duchenne (DMD) patients one or more exons are missing (here a deletion of exon 50), which cases a frame-shift on mRNA level (exon 49 does not fit to exon 51). No functional dystrophin is produced. C. In Becker (BMD), exons are deleted (here a deletion of exon 50–51), but the reading frame is maintained (exon 49 fits to exon 52). From these mRNAs, shorter, but partially functional dystrophins are produced. D. In Duchenne transcripts, ASOs can target a specific exon (here exon 51), thus preventing its inclusion into the mRNA. Thus the reading frame is restored and a DMD patient can make a BMD-type, partially functional dystrophin.

Dystrophin is a structural protein and interestingly the binding domains of the protein are located at the N- and C-terminal parts . The internal domain also has a function, but it is structurally modular with 24 spectrin repeats and 4 hinge regions. As such it is expected that internally deleted dystrophins that do contain the N-and C- terminal binding domains to connect to actin and the proteins linking to the extracellular matrix are partially functional. Indeed, individuals with pathogenic variants that maintain the reading frame cause a less severe muscular dystrophy: Becker muscular dystrophy (BMD) (Figure 2). Without trivializing the impact of BMD, compared to DMD the onset of symptoms is later and disease trajectory is slower, with most patients maintaining ambulation for 10–15 years after the onset of symptoms, which can happen from childhood until middle age.^15^

Most DMD and BMD patients have a deletion variant that removes one or more exons from the DMD gene. Notably, the difference between DMD and BMD is not based on the size of the deletion, but on the reading frame. As such, it is possible to enlarge the deletion for a DMD patient, while at the same time restoring the reading frame. This will then allow the production of a partially functional BMD-type dystrophin. ASOs can target specific exons to cause the skipping and thus ‘re-frame’ transcripts on RNA level (Figure 2). This is a variant-specific approach as different exons need to be skipped depending on the size and location of the deletions. However, as deletions cluster in a hotspot regions between exon 42 and 55, skipping certain exons applies to larger groups of patients, where exon 51 skipping applies to the largest group of patients, ∼14% and therefore ASOs targeting this exons were taken into clinical development first.^16^

## Preclinical and clinical development of nusinersen and eteplirsen

### Nusinersen

For SMA, ASO proof-of-concept was achieved in cell cultures, where exon 7 inclusion was increased after ASO treatment.^17^ A challenge for preclinical development was the fact that only higher primates have *SMN2* genes, while mice do not. Transgenic mouse models with copies of human *SMN2* genes were available. As ASOs do not cross the blood brain barrier, local treatment was required. Unexpectedly, it was revealed that local ASO treatment with an ASO of the methoxyethyl phosphorothioate chemistry into the cerebroventricular space (ICV) in mice or interathecal delivery in nonhuman primates resulted in uptake of ASOs throughout the central nervous system.^18^ Proof-of-concept that ICV injection resulted in increased levels of exon 7 and SMN protein, was achieved in a milder mouse model first.^19^ Later it was also confirmed in a severe SMA mouse model that ICV ASO treatment could improve motorneuron death, motor function and increase survival.^18^

As the ASO targeted mouse models that contained the human *SMN2* gene copies, this ASO (nusinersen), could also be used in humans in clinical trials, using intrathecal delivery. In a placebo-controlled trial in symptomatic type 1 patients the primary endpoint of increased survival (death or permanent ventilation) was met after an interim analysis.^6^ In addition to meeting the primary endpoint, some of the treated patients also achieved milestones never achieved by type 1 SMA patients, including sitting, standing and walking. In a placebo-controlled trial in symptomatic type 2 SMA patients, the placebo group lost motor function over the course of a year, while the nusinersen treated patients gained motor function as measured with the Hammersmith Functional Motor Scale-Expanded.^7^ Based on these findings, nusinersen was granted full marketing authorization by the FDA in December 2016 and by the EMA in April 2017. Currently, nusinersen is approved in over 40 countries and has been used in thousands of patients. Already from the placebo-controlled trial in type 1 SMA patients, it became apparent that the treatment effect was larger when treatment was initiated earlier after onset of symptoms.^6^ It is currently clear that initiating treatment before the onset of symptoms leads to larger therapeutic effects with some treated patients having normal milestone developments.^20^

While intrathecal treatment is invasive, preclinical studies revealed that the ASO and treatment effects persisted for months in the central nervous system after local treatment.^21^ The dosing regimen for nusinersen in patients currently involves 4 loading treatments, where the first 3 are given at fortnightly intervals, and the 4^th^ 1 month later, followed by maintenance doses every 4 months. Each dose consists of 12 mg nusinersen.

### Eteplirsen

For DMD, proof-of-concept was provided in parallel by different groups around the world showing exon skipping and dystrophin restoration in cultured myogenic cells from Duchenne patients and the *mdx* mouse model.^22–24^ Compared to SMA, the DMD field used to believe their clinical ASO development was easier, as systemic treatment was possible to treat skeletal muscle, and it was shown that dystrophic muscle takes up more ASO than healthy muscle.^25,26^ However, skeletal muscle also involves challenges, like its abundance and the fact that the human body has hundreds of skeletal muscles that each require treatment. The *mdx* mouse was instrumental in the preclinical development to obtain proof-of-concept in vivo for dystrophin restoration and functional and histological improvement of skeletal muscles after systemic treatment with exon skipping ASOs.^27^ However, these studies were done with ASOs targeting mouse dystrophin exon 23, which do not apply to a single DMD patient, due to species variation. Furthermore, the dystrophin generated after exon 23 skipping in the *mdx* mouse, differs from the dystrophins generated in DMD patients, who mostly will miss exons in the hotspot region (exon 42–55). A mouse model with the complete human *DMD* gene integrated in the mouse genome was available to test human specific ASOs. However, in this model lack of mouse dystrophin is compensated for by the production of the human dystrophin and therefore this model has no pathology. As such, this model was useful to show exon skipping only.^28^ Mouse models with deletions in both the human and the mouse dystrophin genes have only recently become available.^29^

Clinical trials were initiated with exon 51 targeting ASOs with 2 different chemical modifications, drisapersen (2’-O-methyl phosphorothioate) and eteplirsen (phosphorodiamidate morpholino oligomers). The drisapersen ASO was given by weekly subcutaneous injection and tested in over 300 DMD patients. While there may have been a suggestion for a treatment effect in a subset of younger patients, there were also side effects including injection side reactions in most patients, proteinuria and in a small subset thrombocytopenia. Drisapersen did not receive marketing authorization from FDA and EMA. Eteplirsen, by contrast was tested primarily in open label trials with weekly intravenous injections, which resulted in dystrophin restoration in muscle biopsies albeit at low levels (<1%). In a controversial decision, FDA granted eteplirsen accelerated approval based on the dystrophin restoration (surrogate endpoint), arguing it was reasonably likely that this would lead to future clinical benefit.^2^

A condition of the accelerated approval was that confirmatory studies would be conducted to assess the functional effect of eteplirsen treatment in DMD patients. So far, these results are lacking. A clinical trial using an untreated cohort with different pathogenic variants as a control group failed to show efficacy, as the majority of the untreated cohort did not complete the trial but instead were included in other clinical trials.^30^ There is a growing amount of data on patients who are commercially treated, where there is a suggestion for a treatment effect compared to natural history cohorts.^31^ The confirmatory placebo-controlled trial, MIS51ON is currently ongoing and fully recruited (NCT03992430). Results are expected in 2025.

## Towards the future: What can we learn looking back at the nusinersen and eteplirsen developments?

Looking back, can we retrospectively find success factors that resulted in nusinersen being a treatment where patients achieve milestones they would by definition not achieve without treatment, while for eteplirsen confirmatory data is still forthcoming 8 years post accelerated approval?

A first difference is the fact that nusinersen increases levels of a fully functional protein, while eteplirsen restores production of a partially functional protein. As such, the clinical benefit was always expected to be less for DMD than for SMA. In early exon skipping publications, it was suggested that this would convert a DMD into a BMD phenotype.^22^ However, even that is something the field now realizes is probably an overestimation of the therapeutic effects, as DMD patients will have accumulated muscle damage already at the time of treatment, while BMD patients produce their internally deleted dystrophins from birth.^32^ While for SMA patients it is challenging to quantify the amount of SMN protein produced in the central nervous system due to the inaccessibility of the tissue, post mortem analysis has confirmed protein restoration throughout the brain and the spinal cord.^33^ In DMD patients, dystrophin is restored, but currently the levels are very low.^34^

The main cause for the different efficiencies are the routes of administration that can be used for each of the tissues. For nusinersen local treatment results in a high local exposure of the target cells to the ASO, while for eteplirsen, uptake by skeletal muscle after systemic treatment is very low. Since eteplirsen is small enough to be filtered out by the kidney and is only minimally bound to serum protein, the bioavailability is very limited, and the majority of the compound is excreted via the urine.^35^ Ways to improve skeletal muscle delivery of ASOs are under investigation. For more information we refer the interested reader to a companion paper on this topic in this issue (Arechavala-Gomeza et al., submitted manuscript).

When looking back, one also realizes that the clinical development had different challenges. Both diseases are amongst the most common rare diseases, but for DMD, only a subset of patients was eligible for treatment, which meant trial recruitment was not straightforward. For SMA, the treatment applies to all SMA patients, regardless of the severity type. This complicated trial design as separate trials were needed to assess efficacy for SMA type 1 and type 2. However, for nusinersen establishing clinical efficacy was easier, as patients improved compared to the baseline and even achieved milestones that were clear deviations from the natural history for the SMA type 1. By contrast, as eteplirsen restores the production of a partially functional dystrophin, an improvement compared to baseline is not expected, but rather, a slower decline in disease progression. This is much more challenging to measure, as disease progression in DMD takes years and is not linear for each function.^32^ Since it is not possible to measure a decline in stable patients, this means only certain cohorts can be used to measure a decline in e.g., ambulatory function or upper limb function. As the decline is slow, long clinical trials are required to established that treatment results in a slower decline. This poses challenges on drug developers, clinical trial sites and most importantly on DMD patients and families; e.g., the confirmatory clinical trial for eteplirsen is a 3 year placebo controlled trial with weekly dosing, which is a massive burden. Additional challenges for DMD are the heterogeneity in disease trajectory between patients and the different implementation of the care standards. The use of glucocorticosteroids has been shown to slow down disease progression, but there is also significant heterogeneity with doses and regimen between patients. It is known that disease trajectories differ between patients who are on daily or intermittent regimen.^36^ Another aspect of heterogeneity is that different in-frame dystrophins will be produced after exon 51 skipping depending on the variant (e.g., dystrophins missing the regions coded by exon 45–51, 48–51 or 51–52 for patients with a deletion of exon 45–50, 48–50 or 52).^37^ It is likely that the functionality and the stability of the different dystrophins will vary, and thus the expected therapeutic effect may differ between patients.

There are also similarities: for both diseases a larger therapeutic effect is anticipated when treatment is started earlier. This makes sense as motorneuron loss and loss of muscle tissue are irreversible, so treatment can prevent or slowdown the loss of those motorneurons and muscle fibers that are still there at the time of treatment, but it cannot return the ones that have been lost. For SMA there is also clinical evidence that earlier treatment leads to better results, while for DMD this for now is expected based on pathology and the mechanism of action. For SMA newborn screening initiatives are being set up.^38^ However, it takes years to properly implement this in different countries and during this time a significant number of SMA type 1 patients will only be treated after the onset of symptoms and diagnosis, while they would have benefited more if treatment had started presymptomatically following newborn screening. For DMD, the treatment window is likely slightly larger. However, while symptoms start at age 2–3, pathology starts before this. Following the approval of eteplirsen, newborn screening pilot programs have been initiated in the USA.^39^

Another similarity is that treatments do not treat all the symptoms of the disease. Nusinersen only reaches the central nervous system, while SMN expression is ubiquitous. In mice, body-wide treatment resulted in better and longer term treatment effects than local treatment of the central nervous system.^40^ However, so far there is no evidence that this is the case in SMA patients. For DMD, in animal studies the eteplirsen chemistry was less efficient in the diaphragm, and had negligeable efficiency in the heart at the doses used in the clinic,^41^ while these tissues do also show pathology. Furthermore, eteplirsen does not cross the blood brain barrier, so the brain pathology, which involves cognitive problems,^14^ behavioral and learning difficulties will not be addressed.

Currently, the only way to deliver the ASO systemically and to the CNS is a dual route system, administering the ASOs into the circulation via intravenous or subcutaneous delivery and into the CNS via intrathecal infusions. This would be burdensome to patients. Future developments may circumvent this by allowing ASOs to cross the blood brain barrier efficiently after systemic treatment. At the moment, efforts in animal models show this is feasible, but only when very high doses of ASOs are used,^42^ which would risk e.g., liver and kidney toxicity.

Finally, both drugs are very expensive, with prices at hundreds of thousands of dollars per patient per year. Eteplirsen so far is only approved in a few selected countries, but for nusinersen, which is approval in over 40 countries, this means that access to this treatment depends on the availability of a good national health care system, a good health insurance or family wealth. As such, access is not equal globally.

## Towards the future

Looking back, the authors feel that the main success factor for nusinersen have been that local treatment was feasible and that this was possible with an infrequent maintenance regimen. Furthermore, the fact that patients improved beyond the natural history made clinical development much easier than for DMD, where long trials are needed to show a slower disease progression. For both diseases, gene therapy approaches have also been approved. For SMA, this restores the production of a functional SMN protein, while for DMD again only a partially functional micro-dystrophin is produced. Also for these gene therapy approaches, obtaining clinical evidence of efficacy has been easier for SMA, where SMA type 1 patients achieved motor milestones, than for DMD, where functional efficacy data is still lacking.^43,44^

For DMD, efforts are ongoing to clinically develop ASOs targeting other dystrophin exons and to improve ASO chemistry and delivery to skeletal muscle. We again refer the interested reader to a review paper on this topic in this issue (Arechavala-Gomeza et al., submitted manuscript). With DMD and SMA being rare diseases, however, clinical development of future, improved therapies is becoming increasingly challenging from a clinical trial recruitment perspective. This is especially pertinent for DMD, where multiple clinical developments are ongoing for exon 51, which only applies to a subgroup of patients, and where patients may already have been treated with gene therapy or may be treated commercially with eteplirsen.^45^

Towards the future, more work will be needed as these treatments are not (always) cures. While there are reports of SMA patients who respond very well to nusinersen treatments, there are also patients who respond only partially. The field will have to monitor these individuals closely and find ways to best manage this ‘new type of SMA patients’. For DMD, only partially functional dystrophins are produced so pathology will still happen, just at a lower speed. As such, combination treatments with compounds that slow down pathological pathways are probably require to have a more optimal treatment effect.

## Disclosures

ST and AAR are associate editors for JND, but were not involved in any part of reviewing this work.

For the past 5 years, AAR discloses being employed by LUMC which has patents on exon skipping technology, some of which has been licensed to BioMarin and subsequently sublicensed to Sarepta. As co-inventor of some of these patents AAR was entitled to a share of royalties. AAR further discloses being ad hoc consultant for PTC Therapeutics, Sarepta Therapeutics, Regenxbio, Dyne Therapeutics, Lilly, BioMarin Pharmaceuticals Inc., Eisai, Entrada, Takeda, Splicesense, Galapagos, Sapreme, Italfarmaco and Astra Zeneca. In the past 5 years ad hoc consulting has occurred for : Alpha Anomeric. AAR also reports being a member of the scientific advisory boards of Eisai, Hybridize Therapeutics, Silence Therapeutics, Sarepta therapeutics, Sapreme and Mitorx. SAB memberships in the past 5 years: ProQR. Remuneration for consulting and advising activities is paid to LUMC. In the past 5 years, LUMC also received speaker honoraria from PTC Therapeutics, Alnylam Netherlands, Italfarmaco and Pfizer and funding for contract research from Sapreme, Eisai, Galapagos, Synaffix and Alpha Anomeric. Project funding is received from Sarepta Therapeutics and Entrada via unrestricted grants. Again, for the past 5 years, ST discloses being employed by NCNP which has patents on exon skipping technology and drugs. As co-inventor of some of these patents ST was also entitled to a share of royalties. ST further discloses being ad hoc consultant for Nippon Shinyaku, Taiho, and Daiichi-Sankyo. ST also reports being a member of the scientifc advisory boards of Nippon Shinyaku. ST received speaker honoraria from Nippon Shinyaku and project funding from Taiho.
